# Case report: DKRd regimen in the treatment of newly diagnosed POEMS syndrome and literature review

**DOI:** 10.3389/fonc.2024.1417380

**Published:** 2024-08-01

**Authors:** Jianchao Wang, Wensheng Liao, Zhongwen Liu, Dai Kong

**Affiliations:** ^1^ Department of Surgery of Spine and Spinal Cord, Henan Provincial People’s Hospital, Zhengzhou University People’s Hospital, Zhengzhou, China; ^2^ Department of Hematology, Henan Provincial People’s Hospital, Zhengzhou University People’s Hospital, Zhengzhou, China

**Keywords:** POEMS syndrome, treatment, daratumumab, carfilzomib, lenalidomide, dexamethasone

## Abstract

POEMS syndrome, characterized as a rare multisystem paraneoplastic syndrome, arises from plasma cell abnormalities. Coined by Bardwick in 1980, the acronym POEMS delineates the distinctive features of the syndrome: Peripheral nerve Lesions, Organomegaly, Endocrinopathy, Monoclonal gammopathy, and Skin changes. The prevalence of POEMS syndrome stands at approximately 0.3 per 100,000 individuals. Owing to its low prevalence and the paucity of prospective studies, current treatment approaches largely hinge on retrospective studies and revolve around the use of plasma cell-directed therapy typically used in multiple myeloma treatments. This article presents the pioneering case of utilizing a four-drug combination regimen of DKRd (daratumumab, carfilzomib, lenalidomide, and dexamethasone) as a first-line treatment. This is succeeded by induction therapy and subsequently, autologous hematopoietic stem cell transplantation. A comprehensive review of related literature is conducted.

## 1 Introduction

POEMS syndrome, characterized as a rare multisystem paraneoplastic syndrome, arises from plasma cell abnormalities. Coined by Bardwick in 1980 (1), the acronym POEMS delineates the distinctive features of the syndrome: Peripheral nerve Lesions, Organomegaly, Endocrinopathy, Monoclonal gammopathy, and Skin changes. The prevalence of POEMS syndrome stands at approximately 0.3 per 100,000 individuals (2). Owing to its low prevalence and the paucity of prospective studies, current treatment approaches largely hinge on retrospective studies and revolve around the use of plasma cell-directed therapy typically used in multiple myeloma treatments. This article presents the pioneering case of utilizing a four-drug combination regimen of DKRd (daratumumab, carfilzomib, lenalidomide, and dexamethasone) as a first-line treatment. This is succeeded by induction therapy and subsequently, autologous hematopoietic stem cell transplantation. A comprehensive review of related literature is conducted.

## 2 Case presentation

A 29-year-old male developed numbness in his lower extremities without any apparent triggers. No evident upper extremity weakness, limb pain, or other discomforts were observed, hence no subsequent diagnosis or treatment was administered. two months later, the patient’s symptoms of numbness and weakness in his lower extremities intensified. This was accompanied by numbness in the thumbs and index fingers of both hands, inhibiting his ability to walk normally. He denied any history of trauma, back pain, or high-risk behavior.

**Figure 1:**
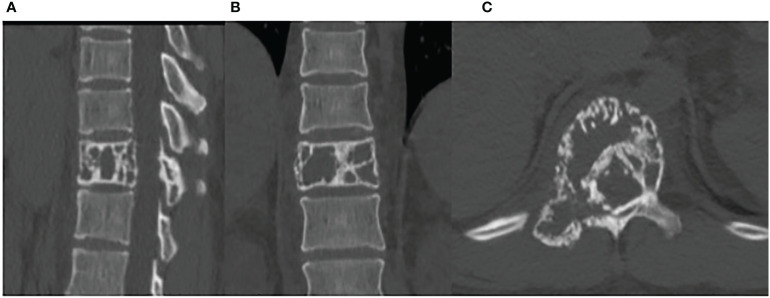
The CT demonstrates bone lesions located in the T11 vertebral body. **(A)** sagittal image. **(B)** coronal image. **(C)** axial image.

**Figure 2:**
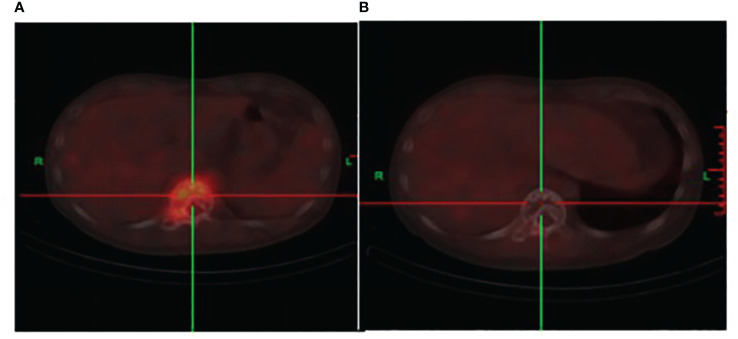
**(A)** The initial 18F-FDG co-registered imaging depicts bone destruction in the 11th thoracic vertebra. An occurrence of soft tissue shadows and an increase in metabolism are also observed. **(B)** After two cycles of DKRd treatment, the 18F-FDG displays bone destruction shadows in the 11th thoracic vertebra, with no significant signs of hypermetabolism, the metabolic shadow is disappearance.

A CT scan showed erosive bone destruction in the patient’s T11 vertebral body (**Figure 1**). The 18F-FDG examination performed high-density nodular shadow in the 11th thoracic vertebra and right accessory bone. Increased metabolic activity indicated potential malignant lesions (**Figure 2**).

**Figure 3:**
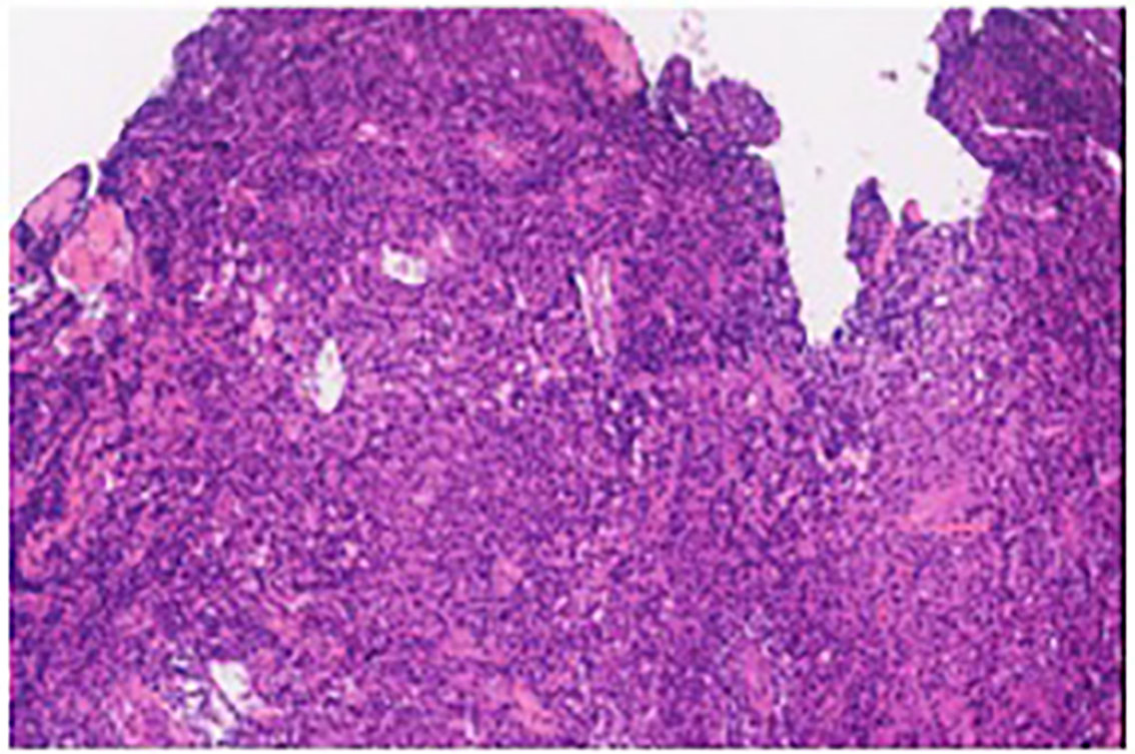
The pathologic immunohistochemistry of the 11th thoracic vertebra lesions is consistent with plasma cell neoplasms.

Biopsy of the spinal mass was as follows: Ki67 (about 5%), CD56 (positive), CD138 (positive), CD38 (positive), Cyclin-D1 (negative), EMA (positive), Mum-1 (positive), Kappa (trace), Lambda (abundant), CD20 (negative), CD79a (positive), CD3 (negative), CD5 (negative), CD19 (negative), CD117 (negative), EBER (negative), P53 (negative), CD21 (negative). These findings are consistent with a diagnosis of plasma cell tumors (**Figure 3**). In the bone marrow cytology examination, no obvious abnormalities were found. Bone marrow immunophenotyping showed 0.43% plasma cells expressing CD38, CD138, CD81and Lambda. Serum immunofixation showed immunoglobulin G was positive. (11.29mg/L (range 8.6–17.4mg/L), and vascular endothelial growth factor (VEGF) was elevated 247.26pg/ml (normal 0–160pg/ml), as was Prolactin at 22.18ng/mL (range 3.90–21.23ng/mL). The patient’s light chain test results: sFLC-λ110.00mg/L(8.3–27)、sFLC-κ32.90mg/L(6.7–22.4)、sFLC-κ/λ 0.30(0.31–1.56)、uFLC-λ75.50mg/L(0–11.3)、uFLC-κ97.30mg/L(0–25.8)、uFLC-κ/λ 1.29(1.4–6.2). Additionally, a reduced level of Vitamin B12 was noted, measured at 105pg/mL (range 180–914pg/mL). The cerebrospinal fluid examination showed a positive Paneth’s test. Protein level was significantly elevated at 2.09 (range 0.15–0.45g/L) and white blood cell count was 5×10^6/L (range up to 8×10^6/L).

A Color Doppler ultrasound of the bilateral breasts and axillary lymph nodes revealed the presence of gynecomastia and Bilateral axillary lymph node cortical thickening. Nerveconduction study showed symmetrical demyelination sensorimotor polyneuropathy while electromyogram showed neuropathic changes.

The DKRd treatment regimen commenced on diagnosis of POEMS syndrome was consequently confirmed. This includes administering Daratumumab on a 28-day cycle at 16 mg/kg once per week during the first 8 weeks, totaling 8 administrations; The drug Carfilzomib was administered on days 1, 2, 8, 9, 15, and 16 of each 28-day cycle. The initial dose was set at 20 mg/m^2^ and was well-tolerated by the patient. On the 8th day of the first cycle, the dose was escalated to 27 mg/m^2^. Lenalidomide was also administered in each 28-day cycle, with a daily dose of 25 mg taken orally for 21 consecutive days. Dexamethasone was administered at a dose of 30 mg/d on the specific days of each 28-day cycle: 1, 2, 8, 9, 15, 16, 22, and 23. The assessment was conducted before the start of the second DKRd cycle. Upon index review and immunofixation electrophoresis test, the results showed IgG at 4.35g/L (range 8.6–17.4)g/L, indicating the presence of IgG-type monoclonal immunoglobulinemia. A significant reduction was noted in the serum vascular endothelial growth factor, measured at 42.35 pg/ml (range 0–160 pg/ml), compared to the initial diagnosis. The patient reported relief from symptoms of lower limb weakness, however, the numbness experienced in the distal limbs demonstrated no significant improvement. An anindexed re-examination and an immunofixation electrophoresis test were scheduled to be conducted following the second cycle of DKRd. The IgG was measured at 3.44g/L (range 8.6–17.4g/L), with no abnormal bands signaling monoclonal immunoglobulin. At the time of initial diagnosis, the patient’s liver SUVmax was 3.11, and the lesion SUVmax was 4.91. Upon follow-up, the patient’s liver SUVmax was 2.85, and the lesion SUVmax was 1.64.The serum vascular endothelial growth factor was found to be 50.63 pg/ml (range 0–160 pg/ml). The 18F-FDG reexamination showed signs of bone lesions in the 11th thoracic vertebra without significant hypermetabolism, and the contrast agent hypermetabolism shadow disappeared (**Figure 2**). Cerebrospinal fluid examination revealed a positive Pan’s test, with the protein level standing at 1.432 g/L (range 0.15–0.45g/L), and the white blood cell count was measured to be 7×10^6/L (range up to 810^6/L). Bone marrow cytology examination and flow immunophenotyping did not identify any abnormalities. Compared to the initial diagnosis, the patient’s lower limb weakness has significantly improved, but there were no significant changes in the numbness of the extremities.

After the DKRdregimen was treatment 3 months, autologous hematopoietic stem cell mobilization was facilitated using CTX3g/m^2^, resulting in the collection of a total of 5.03×10^6^/kg CD34+ cells and 7.1×10^8^/kg MNC. one month later, an autologous hematopoietic stem cell transplant was undertaken, following a pretreatment plan comprising melphalan 200mg/m^2^. Hematopoietic reconstitution proceeded smoothly with NE+14d and PLT+16d engraftment. The patient consistently tested negative in immunofixation electrophoresis, in accordance with regular monitoring and follow-ups. Post-autologous hematopoietic stem cell transplantation, maintenance treatment will commence three months later; it constitutes a 28-day cycle of lenalidomide, at 25 mg/d, administered orally for 21 consecutive days.

## 3 Discussion

Not all five characteristic alterations of POEMS syndrome need to be present at the time of diagnosis, and certain vital disease-related features, like papilledema and vasculature, are excluded from the POEMS acronym. Features such as external volume overload, sclerosing bone lesions, thrombocytosis/erythrocytosis, elevation in VEGF levels, thrombophilia, and anomalous pulmonary function tests are also observed (3). To date, the precise pathogenesis of POEMS syndrome remains unclear, however, it is hypothesized to be linked to an imbalance in cytokine secretion. The overproduction of various pro-inflammatory factors (such as interleukin 1, interleukin 6, fibroblast growth factor, Hepatocyte growth factor, etc.) and VEGF occurs, whereas the synthesis of anti-inflammatory cytokines (like transforming growth factor1) is diminished (4). Specifically, a substantial rise in VEGF is believed to be associated with the characteristic symptoms (5). Unfortunately, the therapeutic efficacy of the VEGF inhibitor, Bevacizumab, seems less than ideal. VEGF is considered not to be the driving factor of POEMS syndrome but rather a downstream mediator of the condition (6). As of this moment, there are no recognized molecular or genetic risk factors that can forecast overall survival. The number of POEMS features present at diagnosis does not serve as a prognostic factor (7). An evaluation of endocrine test outcomes in patients diagnosed with POEMS syndrome revealed that individuals with clinical hypothyroidism exhibited lower rates of progression-free survival and overall survival (8). Patients presenting with Castleman disease demonstrated a lower overall survival rate compared to those without the disease (9). The research by Wang C et al. (10) proposed a clinical predictive model asserting that factors like age over 50 years, presence of pleural effusion, eGFR less than 30 mL/min/1.73 m^2^, and pulmonary hypertension are correlated with poor overall survival rate.

The current approach to treating POEMS syndrome involves the application of antiplasma cell drugs that are typically used for treating multiple myeloma. Radiation therapy serves as the primary line of treatment for patients presenting no more than three bone lesions and monoclonal plasma cells that do not compromise bone marrow. Research conducted by Kourelis TV et al. (11, 12) documented that out of 291 patients diagnosed with POEMS syndrome, 91 persons fulfilled the criteria for radiotherapy. Post-radiotherapy, the 6-year progression-free survival rate stood at 62%, while the 10-year overall survival rate was 70%. Once monoclonal plasma cells compromise the bone marrow, regardless of a significantly low percentage of plasma cells, the disease remains incurable by radiation therapy, necessitating systemic therapy.

Even though patients with POEMS syndrome carry a lower burden of monoclonal plasma cells in comparison to those with multiple myeloma, the deployment of induction chemotherapy preceding autologous hematopoietic stem cell transplantation can nonetheless enhance patient outcomes. Achieving a complete VEGF response (CR_V_) and complete hematological response (CR_H_) prior to autologous hematopoietic stem cell transplantation was found to be associated with improved 5-year progression-free survival (PFS). This association was markedly significant among patients who achieved both CR_V_ and CR_H_ (95% vs 61%, p = 0.004), and it was observed that induction therapy aimed at reducing VEGF levels has the potential to decrease the risk associated with autologous transplantation. However, to counteract the potential risk of impairing stem cell collection due to the onset of related complications and adverse reactions (13), it is advised to abstain from induction therapy regimens that contain melphalan. According to a study conducted by Kourelis TV et al. (11), eligible patients for transplantation are subjected to induction chemotherapy, subsequently followed by autologous hematopoietic stem cell transplantation, utilizing melphalan at a dosage of 140–200 mg/m^2^ as a conditioning regimen. It was observed that 80 participants suffering from POEMS syndrome underwent an autologous hematopoietic stem cell transplantation. The study further revealed a six-year Progression-Free Survival (PFS) rate of 72% and a ten-year Overall Survival (OS) rate of 89%. For patients inhale to undertake autologous hematopoietic stem cell transplantation, consideration may be given to anti-plasma cell therapy. The clinical response rate of the melphalan combined with dexamethasone regimen was reported to be 44% (7). The thalidomide-dexamethasone regimen can lower VEGF levels and extravascular volume overload (14), but studies indicate that thalidomide carries the risk of escalating peripheral neuropathy, thereby limiting this regimen’s clinical application (15). As a novel immunomodulator, lenalidomide exerts an anti-VEGF effect, and it bears significantly lesser neurotoxicity than thalidomide. The CR_H_andCR_V_rates of the lenalidomide-dexamethasone regimen stand at 46%-48% and 43%-48%, respectively. The three-year PFS and OS rates achieve 65%-75% and 83%-90% respectively (16, 17).There is limited data on pomalidomide, which is more commonly seen in patients intolerant to lenalidomide, as case report (18). Bortezomib has been demonstrated to exhibit an anti-VEGF effect. A study conducted by He H et al. (19) involved a sample of 20 patients diagnosed with POEMS syndrome. These individuals were subjected to a regimen of cyclophosphamide, reduced bortezomib, and dexamethasone over 3–6 cycles. The outcomes revealed CR_H_ at 41% and CR_V_ at 76%.According to a report (20) that examined 69 cases undergoing first-line treatment with a combination of bortezomib and dexamethasone, the median number of cycles was 9 (range 1–9) with a CR_H_ of 46%, a CR_V_ of 71%, and a two-year OS of 96%. Furthermore, 3% of these patients developed reversible grade 1 peripheral nerve injuries induced by bortezomib after six treatment cycles, which subsided post- discontinuation. Given that bortezomib can lead to peripheral neuropathy, the use of carfilzomib bears potential for patients diagnosed with POEMS syndrome. In a report by Vaxman I et al. (21), six patients were administered carfilzomib. This treatment regimen, which included Kd, KRd, and KPd (two cases each), resulted in five patients (83%) responding to treatment, three of whom achieved CR/VGPR_H_(very good partial hematological response), one obtaining PR_H_ (Partial Hematological Response), and one achieving clinical symptom remission. Despite the convenience of its oral dosage form, ixazomib’s efficacy in treating POEMS syndrome is not fully substantiated. Dispenzieri A et al. (22) reported the use of a combination of ixazomib, lenalidomide, and dexamethasone for POEMS syndrome. Among the eleven patients with newly diagnosed or recurrent POEMS syndrome, while a CRv of 72% was observed, neuropathy worsened in five of these patients, three experienced disease progression, and two succumbed to the disease. Data on the combined use of immunomodulators and proteasome inhibitors is limited. Jurczyszyn A (23) reported on seven patients who were given first-line combination therapy, with 60% achieving CR_H_/VGPR_H_. Currently, usage of the anti-CD38 monoclonal antibody daratumumab has been predominantly documented in case reports. Dima et al. study shows encouraging results with daratumumab monotherapy in ACTH-naive patients with relapsed POEMS syndrome (24). In conjunction with treatment regimens of lenalidomide and dexamethasone, significant hematological, VEGF, and neurological responses were observed in patients who relapsed after autologous hematopoietic stem cell transplantation and first-line treatment (25, 26). The patient discussed in this article was subjected to the DKRd induction treatment regimen. The patient attained CR_V_ following one treatment cycle. Post two cycles, the patient accomplished CR_H_ and a complete response to the 18F-FDG line imaging examination, which coincided with a noticeable improvement in neurological symptoms. Subsequent to this, autologous hematopoietic stem cell transplantation was administered, which was successful without encountering any transplant-related complications.

## 4 Conclusion

Currently, there is no established treatment protocol for POEMS syndrome. Based on this patient’s case, the DKRd combination regimen shows swift effects without manifesting any marked non-hematological or hematological toxicity. In sequence, the successful autologous hematopoietic stem cell transplantation exhibited no noticeable transplant complications, suggesting it is a new potential treatment option that is worth consideration.

## Data availability statement

The datasets presented in this study can be found in online repositories. The names of the repository/repositories and accession number(s) can be found in the article/supplementary material.

## Ethics statement

Written informed consent was obtained from the minor(s)’ legal guardian/next of kin for the publication of any potentially identifiable images or data included in this article.

## Author contributions

JW: Writing – original draft, Writing – review & editing, Data curation. WL: Data curation, Writing – original draft, Formal analysis. ZL: Funding acquisition, Investigation, Writing – original draft. DK: Data curation, Investigation, Writing – review & editing, Writing – original draft.
